# The loss of OPA1 accelerates intervertebral disc degeneration and osteoarthritis in aged mice

**DOI:** 10.21203/rs.3.rs-3950044/v1

**Published:** 2024-02-20

**Authors:** Makarand Risbud, Vedavathi Madhu, Miriam Hernandez-Meadows, Ashley Coleman, Kimheak Sao, Kameron Inguito, Owen Haslam, Paige Boneski, Hiromi Sesaki, John Collins

**Affiliations:** Thomas Jefferson University; Thomas Jefferson University; Thomas Jefferson University; Thomas Jefferson University; Thomas Jefferson University; Thomas Jefferson University; Thomas Jefferson University; Thomas Jefferson University; Johns Hopkins University; Thomas Jefferson University

**Keywords:** Opa1, mitochondrial dynamics, organelles, metabolism, intervertebral disc, nucleus pulposus, articular cartilage, osteoarthritis, osteopenia

## Abstract

NP cells of the intervertebral disc and articular chondrocytes reside in avascular and hypoxic tissue niches. As a consequence of these environmental constraints the cells are primarily glycolytic in nature and were long thought to have a minimal reliance on mitochondrial function. Recent studies have challenged this long-held view and highlighted the increasingly important role of mitochondria in the physiology of these tissues. However, the foundational understanding of mechanisms governing mitochondrial dynamics and function in these tissues is lacking. We investigated the role of mitochondrial fusion protein OPA1 in maintaining the spine and knee joint health in mice. OPA1 knockdown in NP cells altered mitochondrial size and cristae shape and increased the oxygen consumption rate without affecting ATP synthesis. OPA1 governed the morphology of multiple organelles, including peroxisomes, early endosomes and cis-Golgi and its loss resulted in the dysregulation of NP cell autophagy. Metabolic profiling and ^13^C-flux analyses revealed TCA cycle anaplerosis and altered metabolism in OPA1-deficient NP cells. Noteworthy, *Opa1*^*AcanCreERT2*^ mice with *Opa1* deletion in disc and cartilage showed age-dependent disc degeneration, osteoarthritis, and vertebral osteopenia. Our findings underscore that OPA1 regulation of mitochondrial dynamics and multi-organelle interactions is critical in preserving metabolic homeostasis of disc and cartilage.

## INTRODUCTION

Chronic low back pain, associated with intervertebral disc (IVD) degeneration, and knee and hip joint pain, a common sequela of osteoarthritis, are the leading causes of disability in the aging population^1^. In a healthy state, these tissues provide the joint with flexibility and the ability to absorb applied forces. However, with the onset of degenerative disease, these properties are lost, and the balance between cell survival, autophagy, and apoptosis becomes dysregulated leading to altered extracellular matrix (ECM) production, and increased tissue catabolism^2,3^. Nucleus pulposus (NP) cells of the IVD and articular chondrocytes reside in an avascular, nutrient-limited hypoxic environment that is hyperosmotic and express the transcription factor HIF-1α and exhibit limited replication and regenerative capacity^4,5^ Consequently, these primarily glycolytic cells were thought to rely minimally on mitochondria to meet their energetic and biosynthetic demands^5–7^. However, r mounting evidence suggests that mitochondrial dysfunction promotes osteoarthritis development^3^. The deletion of mitochondrial superoxide dismutase 2 exacerbated, whereas overexpression of catalase or peroxiredoxin 3 reduced the severity of age-associated osteoarthritis in mice, implying that mitochondrial ROS generation plays a role in the pathogenesis of osteoarthritis^8–10^. Moreover, we have recently showed that functional mitochondrial networks exist in NP cells (7), and to meet metabolic requirements, NP cells evidence an active mitophagic flux governed by the HIF-1α-BNIP3 axis^6^. Notably, loss of BNIP3 in NP cells resulted in mitochondrial dysfunction, affecting cellular bioenergetics, triggering meta-inflammation, and causing early onset of IVD degeneration^5^ corroborating earlier studies suggesting association of mitochondrial dysfunction with disc degeneration^11^.

As dynamic organelles, mitochondria undergo fission and fusion to control their mass and numbers. This equilibrium is governed by the fission protein DRP1 and the fusion proteins MFN1, MFN2, and OPA1^12^. The fusion of two adjacent mitochondria promotes membrane tubulation and elongation and is a two-step process: outer mitochondrial membrane (OMM) fusion mediated by MFN1 and MFN2 followed by fusion of the inner membrane (IMM) mediated by OPA1^13^. During fusion, mitochondria control stress and energy needs by incorporating components of the damaged organelles (including mtDNA and respiration complexes) to modulate membrane potential, apoptosis, and calcium signaling^14^. The cristae of the IMM are the site of the respiratory chain complexes concerned with ATP synthesis and electron transport^15^. Notably, OPA1 regulates cristae remodeling and apoptosis, independent of mitochondria fusion^16^. Its ablation results in cristae disorganization and release of apoptotic cytochrome c ^17^. Conversely, OPA1 overexpression facilitates the formation and stability of respiratory chain supercomplexes^18^. OPA1 also functions as a sensor of metabolic changes through interactions with IMM solute transporters, facilitating the adaptation of cristae shape and respiration to cellular energy needs. Despite, the increasingly important role of mitochondria in disc and cartilage the foundational understanding of mechanisms governing mitochondrial dynamics and function in these tissues is lacking.

Herein we investigated the role of OPA1 on IVD and cartilage health. We demonstrate that loss of OPA1 in NP cells results in altered mitochondrial and cristae morphology and mass, with compromised autophagy and metabolism. For the first time, we report the importance of OPA1 in maintaining the morphology of several organelles in the NP cells and show that conditional deletion of OPA1 (*Opa1*^*AcanCreERT2*^) causes disc degeneration, osteoarthritis and osteopenia in aged mice. Overall, these findings establish that the OPA1 is a critical factor required for maintaining NP and chondrocyte function and promoting the health and function of the IVD and knee articular cartilage.

## RESULTS

### OPA1 maintains mitochondrial and multi-organelle morphology in NP cells

**Figure 1:**
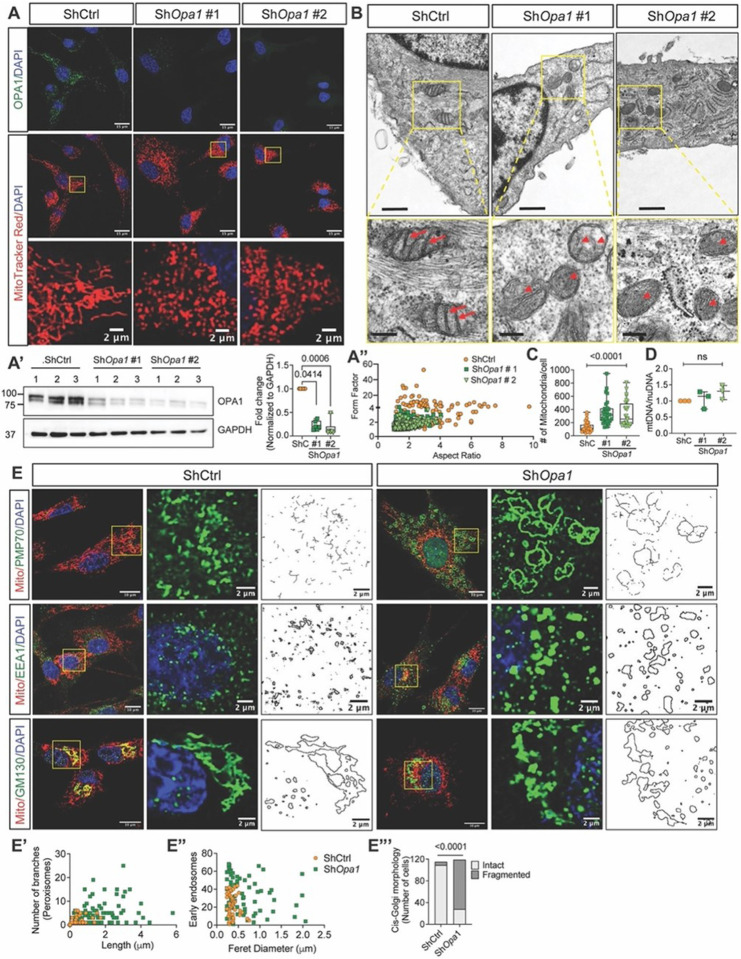
OPA1 maintains mitochondrial and multiple organelle morphology in NP cells. **(A)** Immunofluorescence staining for OPA1 and MitoTracker Red in primary NP cells transduced with lentivirally delivered control (ShCtrl) and *Opa1(ShOpa1* #1 and ShOpa1 #2) ShRNAs. Scale bar: Top rows- 15 μm, bottom row- 2 μm. **(A’)** Western blot to confirm OPA1 knockdown in NP cells. **(A”)** Mitochondrial morphology and network analysis. **(B)** TEM images of ShCtrl and Sh*Opa1* transduced cells. Scale bar: Top row - 600 nm, bottom row - 200 nm **(C)** Mitochondrial number measurements from OPA1-deficient NP cells; 28–30 cells were measured from two independent experiments. **(D)** The mtDNA content in control and OPA1-deficient cells. Data represented as box and whisker plots showing all data points with median and interquartile range and maximum and minimum values. **(E)** Staining for peroxisome marker PMP70, early endosome marker EEA1, and cis-golgi marker GM130. Scale bar: 15 and 2 μm **(E, E”)** Morphology analysis of PMP70, EEA1 shows altered morphology of peroxisomes and early endosomes in Sh*Opa1* cells. **(E’”)** Quantification shows increased fragmented cis-golgi in Sh*Opa1* transduced cells. 100–110 cells were measured for each of the organelle markers. Distribution was performed using chi-square test.

We investigated the contribution of OPA1 in maintaining mitochondrial morphology and mass by knocking down *Opa1* in primary NP cells using lentivirally delivered ShRNAs (Fig. 1A, A’). OPA1-deficient NP cells showed increased mitochondrial fragmentation, evident from the smaller aspect ratio and form factor (Fig. 1A, A”). TEM imaging showed that mitochondria in knockdown NP cells were smaller in size, with absent or aberrant cristae. Although some cristae were often detached, the tubular structure was preserved with narrow cristae width (Fig. 1B). Notably, OPA1-deficient NP cells contained more mitochondria but without changes in mtDNA content, indicating that the increase was due to higher fission and not increased biogenesis (Fig. 1C, D). Moreover, the levels of the outer membrane fusion proteins MFN1, and MFN2 (Fig. S1 A, A’) and the level of the fission protein DRP1 and its receptors FIS1 and MFF were unaffected in ShO*pa1* transduced cells (Fig. S1 A, A’). Together these findings showed that OPA1 deficiency affects mitochondrial number, shape, and cristae morphology without compensatory changes in other fusion and/or fission proteins.

We determined the impact of OPA1 deficiency on the morphology of other organelles considering mitochondrial interactions with many organelles. Peroxisomes are vital for detoxification and β-oxidation of very long-chain fatty acids, which subsequently transports medium-chain fatty acids to mitochondria for further breakdown. Unlike peroxisomes (PMP70 positive) in control cells with lengths between 0.1 to 1 μm, the Sh*Opa1* transduced cells containedhyper-tubulated and hyper-branched peroxisomes that ranged in size from 0.1 to 6 μm (Fig. 1E, E’). We also examined the morphology of EEA1 positive early endosomes, which receive cargo and sort it into recycling and degradative compartments. OPA1-deficient NP cells exhibited enlarged endosomes ranging in size from 0.5–2 μm, as opposed to endosomes that were smaller than 0.7 μm in control cells (Fig. 1E, E”). We also investigated the morphology of the Golgi complex as the primary secretory pathway organelle. There was a pronounced fragmentation of cis-Golgi (GM130 positive) in Sh*Opa1* transduced cells (Fig. 1E, E”‘, Fig. S2A). In contrast, TGN46-stained trans-Golgi, as well as RAB7-stained late endosomes, and LAMP1-stained lysosomes were unaffected (Fig. S2B). These findings suggest that in addition to maintaining mitochondrial and cristae morphology, OPA1 is essential for sustaining the morphology of organelles that include peroxisomes, early endosomes, and the cis-Golgi in NP cells.

### OPA1 deletion disrupts NP cell autophagy

**Figure 2:**
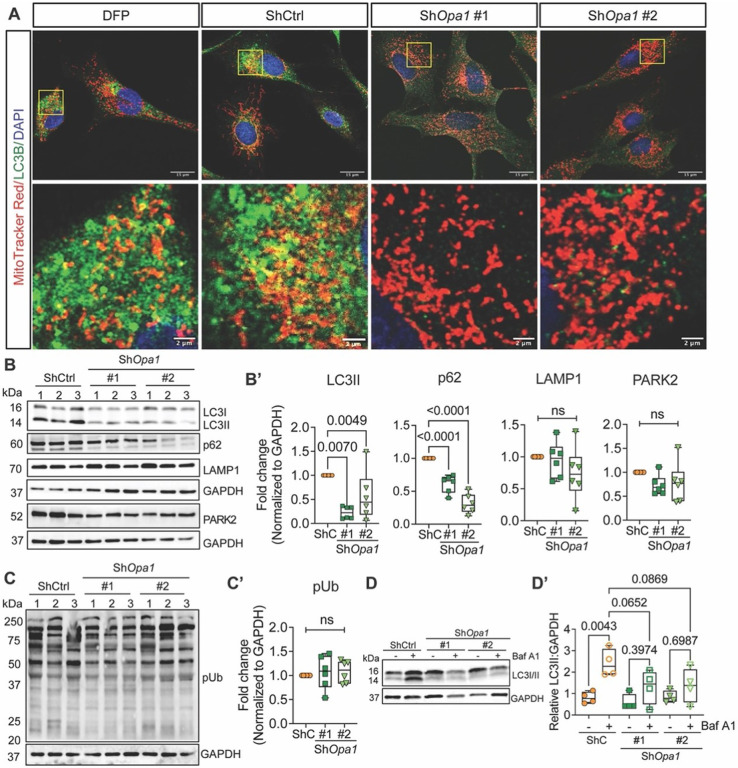
OPA1 deficiency disrupts NP cell autophagy. **(A)** Immunofluorescence staining of LC3B; OPA1-deficient NP cells show a stark absence of LC3B puncta. Scale bar: Top Row-15, Bottom Row - 2 μm. **(B, B’)** Representative Western blot showing data from 3 experiments and densitometric analysis of autophagy/mitophagy pathway markers LC3II, p62, LAMP1, and PARK2 in NP cells sh*Opa1*. **(C, C’)** Representative Western blot and densitometry of phospho-ubiquitin after OPA1 deletion. n = 6 independent experiments. **(D, D’)** Western blot analysis of LC3B in ShCtrl and ShOpa1 transduced cells cultured under hypoxia with or without bafilomycin A1 (n = 4 experiments). Quantitative data represented as box and whisker plots showing all data points with median and interquartile range and maximum and minimum values. Statistical significance was performed using One-way ANOVA or Kruskal Wallis test with Sidaks’s multiple comparisons test as appropriate.

**Figure 3:**
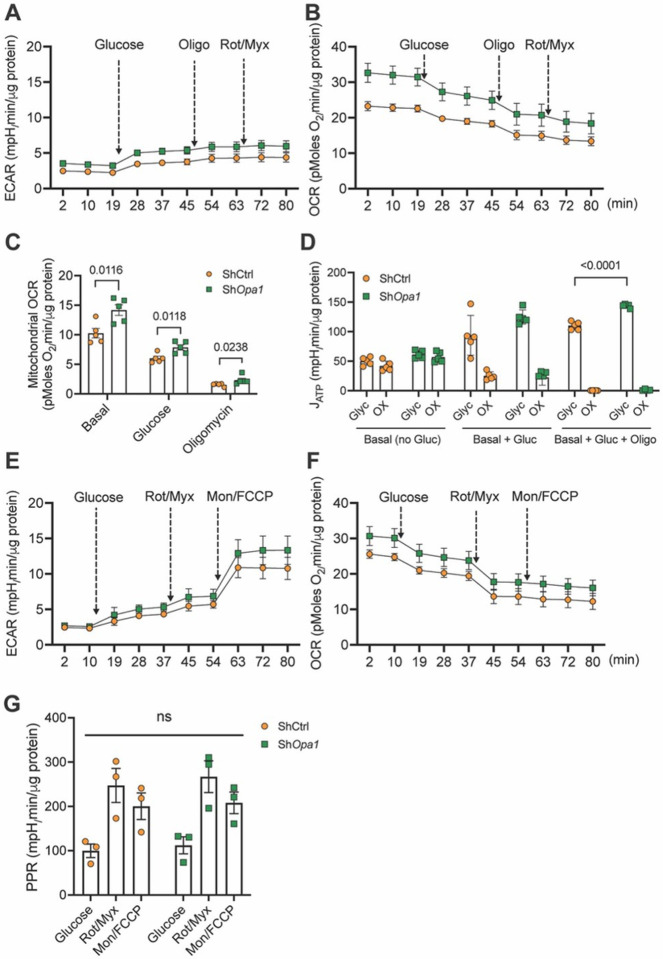
OPA1-deficient NP cells show dysregulated bioenergetics. **(A, B)** ECAR and oxygen OCR traces from NP cells transduced with ShCtrl and Sh*Opa1* in the absence of exogenous glucose and after sequential addition of 10 mM glucose followed by oligomycin and rotenone plus myxothiazol. **(C)** ATP production rate from glycolytic and oxidative metabolism calculated from traces shown in A and B. **(D, E)** ECAR and OCR traces in the absence of exogenous glucose and after sequential addition of 10 mM glucose followed by rotenone plus myxothiazol and finally monensin plus FCCP. **(F)**Proton production rate (PPR) of NP cells calculated from traces shown in D and E. Data represent 3–4 independent experiments each with four replicates/group. Data is shown mean ± SEM. t-test or Mann-Whitney test or one-way ANOVA was used as appropriate.

A striking observation was that OPA1-deficient NP cells showed diminished LC3B-positive autophagosomes (Fig. 2A) while Western blot analysis showed significantly lower LC3II and p62 levels in ShOpa1 cells. These findings suggested an overall reduction in autophagy (Fig. 3B, B’). Since we have recently shown that BNIP3 activation and mitochondrial translocation are required for hypoxia-induced mitophagy in NP cells^6,19^, we investigated whether BNIP3 mitochondrial localization is affected by OPA1 knockdown. Indeed, we observed BNIP3 sequestration in the nucleus of OPA1-deficient cells, without alteration in NIX localization (Fig. S1B, B’) and protein levels (Fig. S1C, C’). The levels of other autophagy-related proteins, BECLIN1, and the ATG12-ATG5 complex remained unaffected whereas those of the apoptotic transcription factor CHOP decreased suggesting Sh*Opa1* cells did not activate apoptosis (Fig. S1C, C’). Moreover, levels of ubiquitinated proteins (Fig. S1D, D’), PARK2, and its substrate phosphoubiquitin, were unaffected by OPA1 knockdown (Fig. 2B, B’, C, C’)^20^. To ascertain whether OPA1 deletion impacts autophagy initiation and/or rate of degradation, we treated cells with bafilomycin A_1_ for 2 hours and noted failure to enhance LC3II accumulation in the Sh*Opa1* cells (Fig. 2D, D’). These data indicate dysfunction in the autophagic pathway of OPA1-deficient NP cells.

### OPA1-deficient NP cells show dysregulated bioenergetics

Since OPA1-deficient NP cells showed mitochondrial fragmentation and abnormal cristae, we measured their ATP production rates from glycolysis and oxidative metabolism by integrating ECAR and OCR measurements (Fig. 3A, B)^5,21^. Under basal conditions, OCR was significantly higher, while ECAR remained unchanged, suggesting OPA1-deficient NP cells consumed more oxygen (Fig. 3A, B, C). Following the addition of glucose, Sh*Opa1* transduced cells showed similar ECAR profiles to ShCtrl group, but the OCR remained significantly higher and similar to the levels observed under basal conditions (Fig. 3C). Further determination of glycolytic ATP generation showed a significant increase in Sh*Opa1* cells; however, oxidative ATP production was comparable between groups despite higher oxygen consumption by OPA1-deficient NP cells. These data suggest uncoupling of OXPHOS in OPA1-deficient NP cells, in conjunction with the aberrant cristae morphology. We also investigated the effect of OPA1-deficiency on the glycolytic capacity of NP cells^5,22^ by measuring ECAR and OCR profiles under basal (no substrate) conditions and following sequential addition of glucose, rotenone + myxothiazol, and monensin + FCCP (Fig. 3E, F). The Sh*Opa1* cells showed no difference in EACR but demonstrated a prominent increase in OCR (Fig. 3E, F). However, the basal glycolytic rate, maximum glycolytic capacity, ATP demand-limited rate, and glycolytic reserve computed from H+/lactate production were unaffected (Fig. 3G). Overall, these results suggest that OPA1-deficiency compromises energy metabolism of NP cells.

### OPA1-deficient NP cells evidence altered glucose and glutamine metabolism

**Figure 4:**
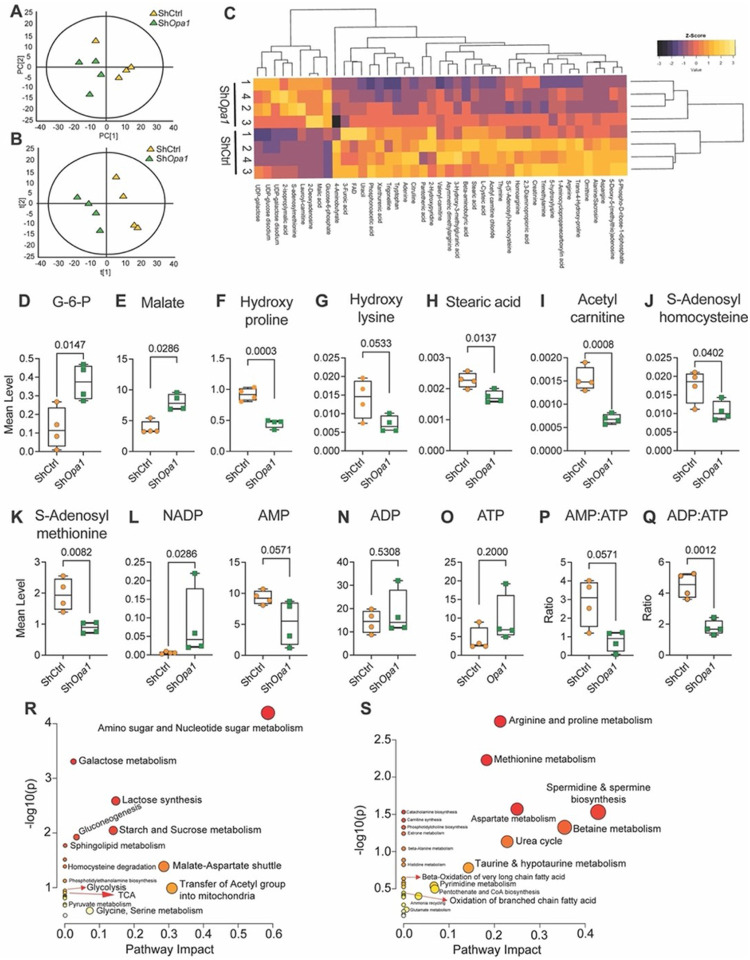
OPA1 is an important regulator of hypoxic NP cell metabolism. **(A)** Unsupervised PCA and **(B)** Supervised PLS-DA model of widely targeted small metabolites from NP cells transduced with ShCtrl and Sh*Opa1*. n = 4 independent experiments **(C)** Heat map of normalized concentration of metabolites differentially present between ShCtrl and *ShOpa1*, FDR <0.05%. **(D–Q)** Mean levels of a select group of measured metabolites from ShCtrl and Sh*Opa1* NP cells **(D)** Glucose-6-phosphate, **(E)** Malate, **(F)** Hydroxyproline, **(G)** Hydroxylysine, **(H)**Stearic acid, **(I)** Acetylcarnitine, **(J)** S-adenosylhomocysteine and **(K) s-**adenosylmethionine, **(L)** NADP, **(M)** AMP, **(N)** ADP, **(O)** ATP, **(P)** ADP/ATP and **(Q)** AMP/ATP. **(R,S)** Metabolite set enrichment analysis (MSEA) showing pathway impact and p-value of significantly upregulated and downregulated metabolites (FDR≤0.05) from Sh*Opa1* transduced NP cells. Quantitative data is represented as box and whisker plots showing all data points with median and interquartile range and maximum and minimum values. Statistical significance was computed using t-test or Mann-Whitney test as appropriate (D–Q).

To understand the broader metabolic implications of OPA1 deficiency, we performed widely targeted metabolic profiling on NP cells. A total of 261 metabolites that satisfied the QC limit of < 30% coefficient of variation (CV) were imported individually into the SIMCA-p program for multivariate analysis. An unsupervised principal component analysis (PCA) and supervised partial least square-discrimination analysis (PLS-DA) models were established which showed a clear separation between the Sh*Opa1* and ShCtrl samples (Fig. 4A, B). We identified 36 downregulated and 9 upregulated metabolites (FDR ≤ 0.05) in OPA1-deficient cells (Fig. 4C). Concerning altered metabolites, the levels of glycolytic metaboliteglucose-6-phosphate and TCA metabolite malate, as well as the collagen-related amino acids hydroxyproline, and hydroxylysine were raised (Fig. 4D–G), whereas fatty acid metabolites stearic acid and acetylcarnitine and the one carbon metabolites s-adenosyl homocysteine and s-adenosyl methionine were significantly lower in OPA1 knockdown cells. (Fig. 4H–K). Additionally, the Sh*Opa1* group showed a significant increase in NADP with lower AMP, without affecting the overall ADP and ATP levels (Fig. 4L–O). However, AMP/ATP and ADP/ATP ratios were affected in knockdown NP cells (Fig. 4P, 4Q). Analysis of upregulated metabolites revealed enrichment in galactose metabolism, nucleotide sugar metabolism, starch, and sucrose metabolism, ascorbate and aldarate metabolism, pentose and glucuronate interconversion, sphingolipid metabolism, pentose phosphate pathway activity, glycolysis/gluconeogenesis, and inositol phosphate metabolism-related metabolites. Similarly, metabolic pathway analysis of upregulated entities showed nucleotide sugar metabolism, malate-aspartate shuttle activity, transfer of acetyl group into mitochondria, and starch and sucrose metabolism, and lactose synthesis were the most impacted pathways in Sh*Opa1* transduced cells (Fig. 4R, Fig.S3A). Arginine, cysteine, methionine, proline, alanine, aspartate, and glutamate were among the enriched metabolites that were significantly downregulated, and the enrichment analysis showed downregulation of aminoacyl-tRNA biosynthesis, pantothenate and CoA biosynthesis, pyrimidine and purine metabolism and taurine and hypotaurine metabolism in Sh*Opa1* cells. Likewise, the most impacted downregulated pathways in Sh*Opa1* cells were spermidine and spermine biosynthesis, and betaine, urea, aspartate, methionine, arginine, and protein, taurine and hypotaurine, pyrimidine, and pantothenate and CoA metabolism, and ammonia recycling (Fig. 4S, Fig. S3B).

**Figure 5:**
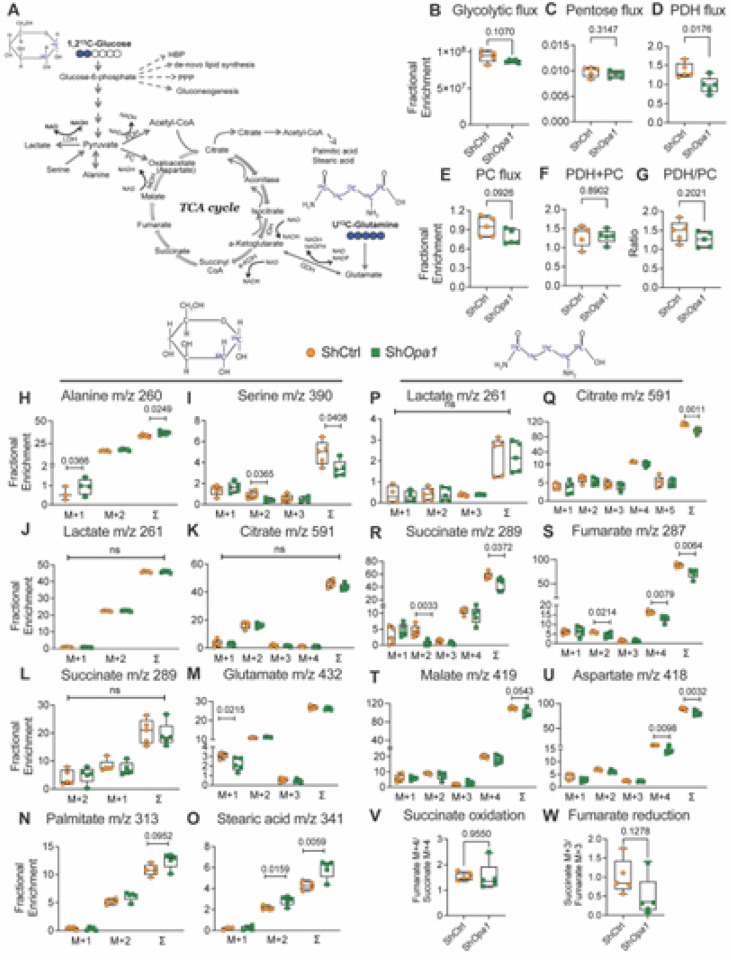
OPA1-deficient NP cells evidence altered glucose and glutamine metabolism. **(A)** Summation of flux results through glycolysis, pentose, and TCA cycle using [1,2]-^13^C-glucose and U^13^C-glutamine. **(B-G)** [1,2]-^13^C-glucose enrichment in the culture media from NP cells transduced with ShCtrl and Sh*Opa1* and cultured under hypoxia for 24 h. **(B)** Glycolysis flux as measured by enrichment of M2 lactate m/z 91, **(C)** Pentose cycle flux as measured by lactate (M1/M2)/ (3 + M1/M2) **(D)** PDH flux as measured glutamate m/z 103, (E) PC flux measured as glutamate m/z 104 **(F)** PC+PDH flux measured as glutamate m/z 131, **(G)** PDH/PC flux as measured by glutamate m/z 103-M1/104-M2. (H-O) [1,2]-^13^C-glucose tracing measured from the cell pellets into metabolites **(H)** alanine m/z 260, **(I)** serine m/z 390, **(J)** lactate m/z 261, **(K)** citrate m/z 591, **(L)** succinate m/z 289, **(M)** glutamate m/z 432, **(N)** palmitate (C16:0) m/z 313, **(O)** stearic acid (C18:0) m/z 341. **(P-W)** U 13C-glutamine tracing measured from NP cells transduced with ShCtrl and ShOpa1 and cultured under hypoxia for 24 h **(P)** lactate m/z 261, **(Q)** citrate m/z 591, **(R)** succinate m/z 289, **(S)** fumarate m/z 287, **(T)** malate m/z 419, **(U)** aspartate m/z 418, **(V)** succinate oxidation and **(W)** fumarate reduction. Quantitative data is represented as box and whisker plots showing all data points with median and interquartile range and maximum and minimum values. Data points with negative values (undetectable ∑ mn) are not included. Statistical significance was computed using t-test or Mann-Whitney test as appropriate.

To further delineate the utilization of major metabolic substrates glucose and glutamine, OPA1-silenced NP cells were cultured for 24 hours under hypoxia with a 50% enrichment in either [1,2]-^13^C-glucose or U^13^C-glutamine (Fig. 5A). Based on the lactate and glutamate of the medium we calculated the glycolytic, pentose cycle, PDH, PC, PDH + PC, and PHD/PC flux, as previously described^5^. OPA1-silencing did not affect glucose flux through glycolysis or the pentose cycle (Fig. 5B, C). ^13^C enrichment in medium glutamate provided a measure of glucose flux into the TCA cycle via PDH and PC. PDH flux was significantly decreased, without altering flux through PC; combined PDH + PC flux or the PDH/PC also remained unchanged (Fig. 5D–G).

Analysis of extracted metabolites from the cell pellet showed a modest increase in sigma mean (∑ mn = 1 *M1 + 2*M2 + 3*M3, etc.) of alanine (m/z 260) and a decrease in serine (m/z 390) with minimal changes in lactate (m/z 261), citrate (m/z 591), and succinate (m/z 289). A small decrease in M1 glutamate (m/z 432) with minimal change in palmitate (m/z 313) as well as enhanced enrichment in stearic acid (m/z 341) was also noted (Fig. 5H–O). When U^13^C-glutamine utilization was measured, Sh*Opa1* cells evidenced a reduction in TCA cycle intermediates citrate (m/z 591), succinate (m/z 289), fumarate (m/z 287), malate (m/z 419), and aspartate (m/z 418), without changes in the enrichment of lactate (m/z 261) (Fig. 5P–U). Both hypoxia and ETC inhibition are shown to increase the levels of succinate^23^. Interestingly, however, our ^13^C-MFA data showed increased succinate ∑ mn compared to fumarate in both ShCtrl and Sh*Opa1* cells, suggesting impairment of the oxidative TCA cycle. Furthermore, when we assessed succinate oxidation (fumarate M + 4/succinate M + 4) and fumarate reduction (succinate M + 3/fumarate M + 3), no change in the oxidation, with a downward trend in fumarate reduction was noted (Fig. 5V, W). These studies lent support to the notion that OPA1 is required for optimal NP cell metabolism.

#### Conditional deletion of Opa1 in IVD accelerates age-associated degeneration

**Figure 6:**
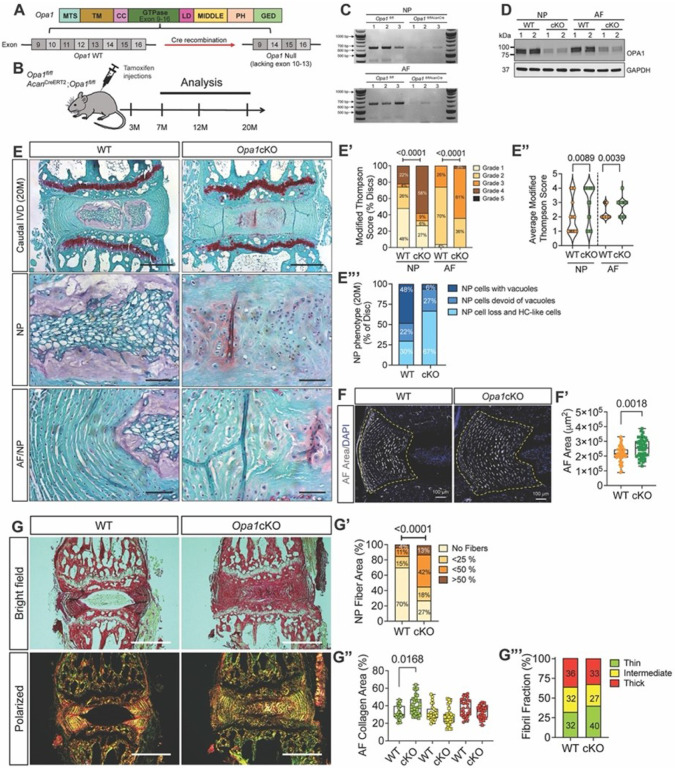
Conditional deletion of *Opa1* in IVD accelerates age-associated degeneration. **(A)** Schematic showing tamoxifen induced *Acan*^*CreERT2*^ mediated deletion of exons 10–13 of *Opa1* to generate *Opa1* null allele. **(B)** Tamoxifen treatment and analysis timeline of WT (*Opa1*^fl/fl^) and *Opa1*^*AcanERT2*^ (Opa1cKO) mice. **(C)** RT-PCR analysis shows *Opa1* deletion in NP and AF tissues of *Opa1cKO* mice (n=3 mice/genotype). **(D)** Western blotting of OPA1 from NP and AF tissues (n=2 mice/genotype). **(E)** Representative Safranin O/Fast Green/Hematoxylin staining of 20-month-old WT and *Opa1cKO* caudal disc sections. Scale bar: Top row-500 μm, Bottom rows - 100 μm. **(E’, E”)** Modified Thompson Scores of NP and AF compartment of WT and *Opa1cKO* caudal discs. **(E’”)** Distribution graph showing NP cell phenotype at 20-months. n = 9 WT **(4F, 5M)**, 11 *Opa1cKO* (6F, 5M) mice; 3 caudal discs/animal, 27–33 discs/genotype. **(F, F**^**’**^**)** AF tissue hyperplasia in *Opa1cKO* caudal discs, dotted lines demarcate AF compartment. scale bar = 100 μm. **(G)** Representative Picrosirius red stained brightfield and polarized light images of caudal discs from 20-month-old mice showing NP fibrosis. **(G’)** The distribution of discs based on %NP area occupied by collagen fibers. **(G”, G’”)** Thin, intermediate, and thick AF collagen fibril fraction. n = 9–11 mice/genotype; 3 caudal discs/animal, 27–33 discs/genotype. Significance for E’, E”, G’, G’” was determined using a chi-square test. The significance of the fiber percentage of AF collagen area **(G”)** was determined using Kruskal-Wallis with Dunn’s test. Quantitative Data represents violin **(E’)** or box and whiskers **(F’, G”)** plot showing all data points with median and interquartile range and maximum and minimum values. Significance was determined using an unpaired t-test or Mann-Whitney test, as appropriate.

To gain a better understanding of how OPA1 impacts spinal health, we generated *Opa1* conditional knockout mice by administering tamoxifen to 3-month-old Acan^*CreERT2*^*Opa1*^*fl/fl*^ *(Opa1cKO)* and *Opa1*^*fl/fl*^ (WT) mice (Fig. 6A, B)^24^. In adult mice, the Acan-Cre^ERT2^ driver is highly effective in targeting all three compartments of the IVD as well as the articular and growth plate cartilages^25^. The successful deletion of OPA1 in IVD was confirmed by mRNA and protein evaluation of the NP and annulus fibrosus (AF) tissues (Fig. 6C, D). We performed a quantitative histopathological analysis of IVD morphology using the Modified Thompson grading on 7, and 12-month-old *Opa1cKO* mice (Fig. S4) which indicated no noticeable degeneration in the IVD in the lumbar (Fig. S4. A-A”, B-B”) or caudal regions of the spine (Fig. S4. C-C”, D-D”). At 12 months, however, caudal discs of *Opa1cKO* mice showed changes in NP cell morphology and AF hyperplasia (Fig. S4D”‘). When *Opa1cKO* mice were evaluated at 20 months, a prominent degenerative phenotype in the NP and AF tissues of caudal IVDs compared to WT mice was evident (Fig. 6E). The distribution of Modified Thompson grading scores showed a significantly higher proportion of *Opa1cKO* discs had NP and AF compartments scores of 3 or 4, indicating severe degeneration (Fig. 6E’, E”). At 20 months, the degenerative phenotype was characterized by a diminished SafraninO stained NP extracellular matrix, a significant loss of NP cells with the remainder of cells acquiring a hypertrophic chondrocyte-like morphology and irregularities in AF lamellar organization (Fig. 6E). There was also a clear loss of demarcation between NP and AF tissue boundaries in *Opa1cKO* mice (Fig. 6E). Those discs that retained notochordal NP cell bands displayed morphological changes such as loss of cytosolic vacuoles (Fig. 6E”‘). In addition to these morphological changes, *Opa1cKO* mice exhibited AF hyperplasia reflected in increased tissue area (Fig. 6F, F’).

Picrosirius red staining coupled with polarized light imaging was used to ascertain alterations in collagen matrix organization (Fig. 6G). At 20 months, *Opa1cKO* mice showed the presence of collagen fibers in the NP compartment. Moreover, when the fraction of fibrous tissue area in the NP was measured; *Opa1cKO* animals also exhibited a higher proportion of fibrous tissue area than a few discs in WT mice with NP fibrosis (Fig. 6G’). In the AF tissue, *Opa1cKO* mice showed a higher proportion of thin collagen fibers suggesting increased turnover (Fig. 6G”, G”‘). Notably, in 20-month-old mice lumbar IVDs showed a milder phenotype compared to caudal discs; there was a higher proportion of lumbar discs with NP and AF compartments scoring grade 4 but when scores of all discs were averaged it did not reach statistical significance (Fig. S5A, A’, A”). Similar to caudal IVDs, the NP compartment of lumbar IVDs in 20-month-old *Opa1cKO* mice displayed a higher prevalence of fibrous tissue underscoring degeneration (Fig. S5B, B’).

### OPA1 deletion affects NP cell phenotype and alters the IVD matrix composition

**Figure 7:**
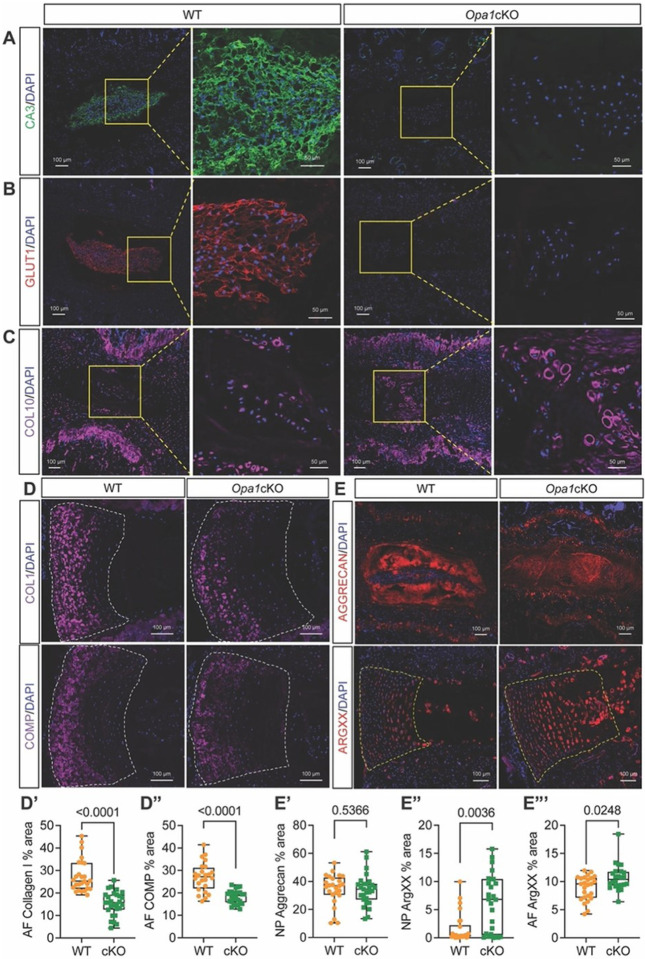
The OPA1-deletion affects NP cell phenotype and alters the IVD matrix composition in mice. Immunohistological staining of 20-month WT and *Opa1*cKO caudal discs for **(A)** carbonic anhydrase 3 (CA3); **(B)** glucose transporter 1 (GLUT1) and **(C)** collagen 10 (COLX). Scale bar = 100 and 50 μm. Immunohistological staining for **(D, D’)**collagen 1 (COL1); **(D, D”)** cartilage oligomeric matrix protein (COMP), **(E, E’)** aggrecan (ACAN); **(E, E”)** aggrecan G1 neoepitope (ARGxx). Scale bar = 100 μm. (n = 9–11 mice/genotype, 1–3 discs/mouse, 13–14 discs/genotype/marker). Quantitative measurements are shown as Box and whisker plots showing all data points with median and interquartile range and maximum and minimum values. Significance was determined using an unpaired t-test or Mann-Whitney test, as appropriate.

We assessed the NP cell phenotype in 20-month-old mice by measuring the abundance of the phenotypic markers, carbonic anhydrase (CA3), and glucose transporter I (GLUT1). Strikingly, expression of these markers was lost by the few cells that persisted in the NP compartment, suggesting the native notochord cell population underwent a phenotypic switch (Fig. 7A, B).

Considering the NP cells may have undergone a phenotypic shift, we stained IVD sections for COLX, a marker of hypertrophic chondrocytes. Staining was considerably higher in both the NP and AF of *Opa1cKO* mice (Fig. 7C), suggesting that resident cells in the NP compartment acquired hypertrophic chondrocyte-like characters. We also noted that *Opa1cKO* had a lower AF abundance of collagen I (COLI) (Fig. 7D, D’) and cartilage-oligomeric matrix protein (COMP), an important non-collagenous matrix component (Fig. 7D, D”). While ACAN staining revealed no major differences across genotypes (Fig. 7E, E’), the cells in the NP and AF compartments of of *Opa1cKO* showed a robust increase in the pericellular staining of ARGxx, an ACAN neoepitope formed by ADAMTS4/5 dependent cleavage (Fig. 7E, E”). Overall, these findings suggest that OPA1 is required for maintaining the IVD cell phenotype and major structural components of the ECM.

#### Opa1 cKO mice evidence alterations in vertebral bone health

**Figure 8:**
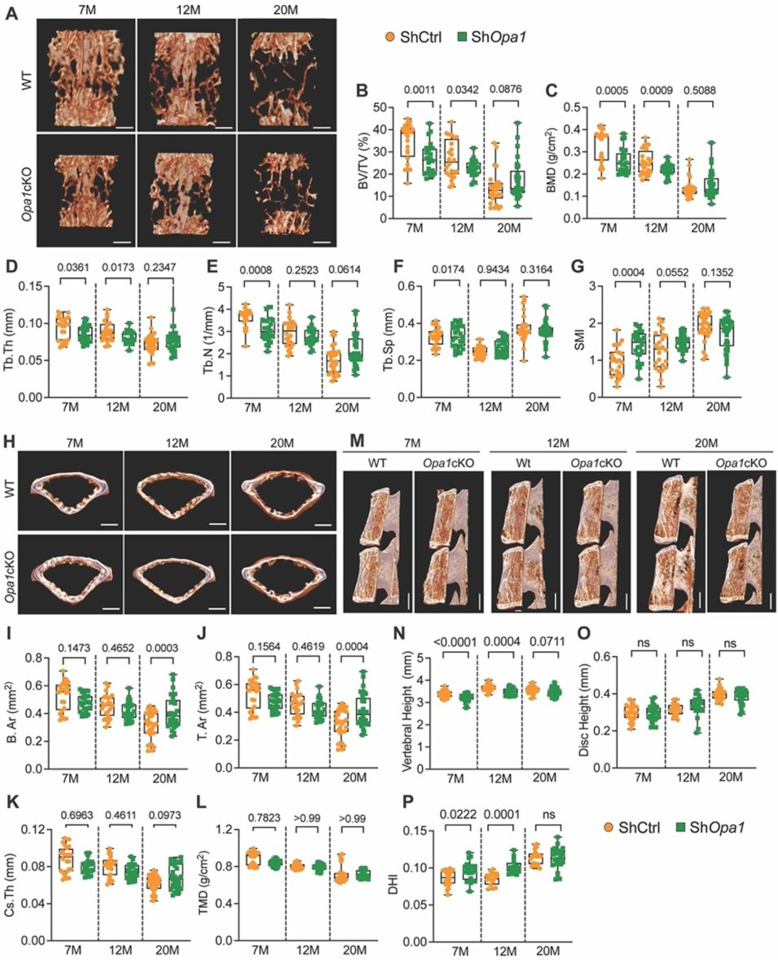
Opa1cKO mice evidence alterations in vertebral bone health and disc height index. **(A)** The representative 3D reconstruction of hemi-section and trabecular lumbar vertebral bone from 7-month, 12-month, and 20- month-old WT and *Opa1*Cko mice. Scale bars 500 μm. Vertebral trabecular bone parameters **(B)** Percent bone volume/ tissue volume (BV/TV) (%), **(C)** Bone mineral density (BMD) (g/cm^3^), **(D)** trabecular thickness (Tb.Th) (mm), **(E)** trabecular number (Tb.N) (1/mm), **(F)** trabecular separation (Tb.Sp) (mm), **(G)** structural model index (SMI) of WT and *Opa1c*KO mice. **(H)** Representative 3D reconstruction of transverse section through vertebrae of WT and *Opa1*cKo mice showing cortical shell geometry and corresponding quantitative parameters **(I)** mean total cross-sectional thickness bone area (B.Ar) (mm2), **(J)** mean total cross-sectional tissue area (T.Ar) (mm^2^), **(K)** cross-sectional thickness (Cs.Th) (mm), **(L)** Tissue mineral density (TMD) (g/cm3). Scale bars 500 μm. **(M)** Representative microCT reconstructions of hemi-sections through vertebrae of WT and Opa1cKO mice to determine **(N)** Vertebral length (VL) (mm), **(O)** disc height (DH) (mm), and **(P)** disc height index (DHI). Scale bars 1 mm. Quantitative data are shown as box and whisker plots showing all data points with median and interquartile range and maximum and minimum values. 7-month n = 10 WT (3F, 7M), 8 *Opa1*cKo (3F, 5M); 12-month n = 10 WT (6F, 4M), 10 *Opa1*cKO (4F, 6M); 20-month n = 9 WT (4F, 5M), 11 *Opa1*cKO (6F, 5M), 4 vertebrae and 3 discs/mouse were analyzed. Significance was determined using t-test or Mann-Whitney test, as appropriate.

To investigate the impact of OPA1 deletion on vertebral bone health we performed micro-computed tomography (μCT) on the lumbar (L3–6) vertebrae of *Opa1*cKO and WT mice. In WT mice, three-dimensional (3D) reconstructions showed expected trabecular bone loss with aging; however, *Opa1cKO* mice exhibited a significant reduction in vertebral trabecular bone architecture at 7- and 12-months (Fig. 8A). Thus, bone volume/trabecular volume (BV/TV), bone mineral density (BMD), trabecular thickness (Tb.Th), and trabecular number (Tb.N) was decreased. The change in bone structure in the 7 and 12-month-old *Opa1*cKO was maintained at 20 months implying early onset osteopenia (Fig. 8B–E). As expected, trabecular separation was also greater in 7-month-old *Opa1*cKO mice (Fig. 8F) and the structural model index (SMI), a parameter that identifies the rod-like structure of trabeculae and is linked to bone strength and fracture risk, was considerably greater at 7 and 12 months (Fig. 8G). Interestingly, unlike early changes noted in trabecular bone of *Opa1cKO* mice, changes in cortical bone structure were evident predominantly at 20-months, with a substantial increase in mean total cross-sectional bone area (B.Ar.), tissue area (T.Ar.) and the cross-sectional thickness (Cs.Th.), without a change in tissue mineral density (TMD) (Fig. 8H–L). We also noted an increased vertebral length and disc height index (DHI) in *Opa1cKO* mice at 7- and 12- months (Fig. 8M–P), which has been correlated with IVD degeneration^26^. These findings suggest that OPA1 plays an important role in the control of vertebral bone health.

#### Opa1 cKO mice show increased severity of age-associated OA

**Figure 9:**
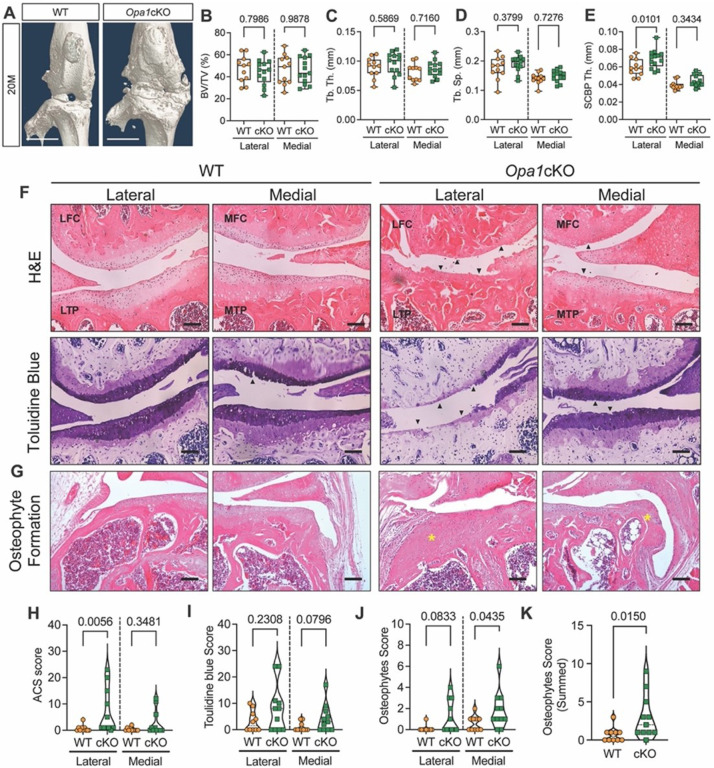
*Opa1*cKO mice show increased severity of age-dependent OA. **(A)** Representative 3D reconstruction of the whole knee joint of 20-month-old WT and *Opa1*cKO mice. Extensive bone spurs are evident in the *Opa1*cKO joint. Scale bar: 500 mm. **(B-E)** Quantitative analyses of tibial subchondral bone parameters on the medial and lateral tibial plateaus of 20-month-old WT and *Opa1*cKO mice (B) bone volume fraction (BV/TV), **(C)**trabecular thickness (Tb.Th), (**D)** trabecular separation (Tb.Sp), **(E)**subchondral bone plate thickness (SCBP.Th). n = 11 WT (4F, 6M); 13 *Opa1*cKO (5F, 8M). **(F)** Representative images of H&E and toluidine blue stained midcoronal sections showing the lateral and medial tibial plateaus and femoral condyles from WT and *Opa1*cKO mice. Extensive cartilage damage (black arrowheads) predominantly in the lateral and medial compartments of *Opa1*cKO mice. Scale bar: 100 μm. **(G)** Representative images of H&E stained sections showing the presence of large osteophytes on the medial and lateral tibial plateaus of *Opa1*cKO mice (yellow asterisks). Scale bar 100 μm. **(H-J)** Summed medial (MTP, MFC) and lateral (LTP, LFC) scores for **(H)** Articular Cartilage structure (ACS), **(I)** Toluidine blue **(J-K)**osteophytes. LFC = lateral femoral condyle, LTP = lateral tibial plateau, MFC = medial femoral condyle, MTP = medial tibial plateau. n = 10 WT (4F, 6M), n = 11 *Opa1*cKO (5F, 6M). Data is represented as box and whiskers (B-E) and violin (H-K) plots showing all data points with median and interquartile range and maximum and minimum values. Significance was determined using t-test or Mann-Whitney test as appropriate.

Since the *Acan*^*CeERT2*^ allele efficiently targets articular cartilage, we investigated if OPA1 deletion has deleterious effects on knee articular cartilage and overall knee joint health in mice. μCT images showed evidence of bone spurs in the knee joints of 20-month-old but not 12-month-old *Opa1cKO* mice (Fig. 9A, Fig. S6A). μCT was also used to evaluate tibial subchondral bone volume fraction (BV/TV), trabecular thickness (Tb.Th), trabecular separation (Tb.Sp), and subchondral bone plate thickness (SCBP) in the medial and lateral tibial plateaus. These analyses showed a significant increase in subchondral bone thickness in the lateral compartment of the 20-month-old mice (Fig. 9B–E); none of the measured bone metrics showed changes at 12 months (Fig. S6B-E). To study cartilage structure, we stained 12- and 20- month-old knee joints sectioned in the mid-coronal plane with H&E and toluidine blue as we have previously described^27^ (Fig. 9F, G). At 20 months, *Opa1cKO* mice showed severe OA with loss of articular cartilage on the lateral tibial plateaus and femoral condyle, but only minor degradation of articular cartilage in the medial compartment (Fig. 9F). Furthermore, H&E staining revealed osteophyte formation in the medial compartment of WT mice, and osteophyte formation in both the lateral and medial compartments of *Opa1cKO* animals (Fig. 9G). When these structural changes were quantified, *Opa1cKO* mice exhibited a significant increase in articular cartilage score (ACS) in the lateral knee compartment at 20-months. However, there were no deviations in toluidine blue scores and osteophyte scores between knee compartments or genotypes (Fig. 9H–J). However, the cumulative osteophyte score in *Opa1*cKO mice was significantly higher than in WT (Fig. 9K).

**Figure 10:**
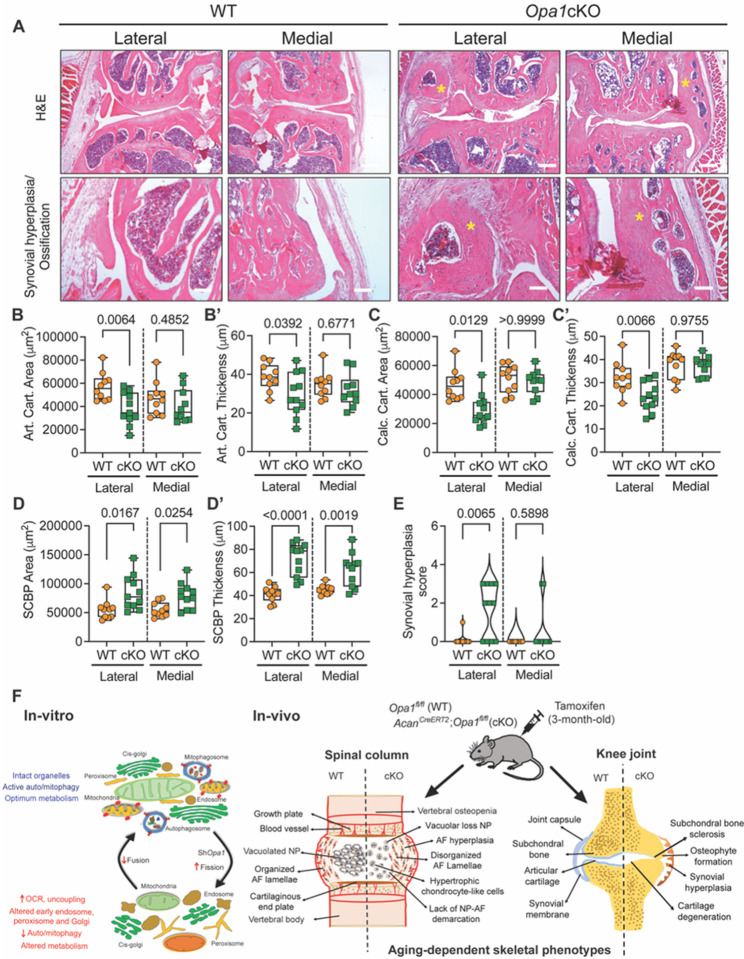
*Opa1*cKO mice evidence synovial hyperplasia and morphological changes in articular cartilage and subchondral bone **(A)** Representative H&E images of WT and *Opa1*cKO mouse joint sections showing medial and lateral compartments. Profound synovial hyperplasia and/or ossification is noted in *Opa1*cKO mice in both the medial and lateral compartments (yellow asterisks), when compared to WT mice. Scale bar: Top row - 250 μm, bottom row - 100 μm. **(B-E)**Histomorphometric analyses of lateral and medial joint compartments conducted on midcoronal sections of WT and *Opa1*cKO mouse limbs. **(B, B’)**articular cartilage (Art. cart) area and thickness, **(C, C’)** calcified cartilage (Calc. cart) area and thickness, and **(D, D’)** subchondral bone plate (SCBP) area and thickness **(E)** quantitative analysis of synovial hyperplasia. n = 10 WT (4F, 6M), n = 11 *Opa1*cKO (5F, 6M). Quantitative data is represented as box and whiskers **(B-D’)** or violin **(E)** plots showing all data points with median and interquartile range and maximum and minimum values. Significance was determined using t-test or Mann Whitney test as appropriate, as appropriate. (F) Schematic showing the in vitro consequences of OPA1-deficiency on mitochondrial and organelle morphology and metabolic functions of NP cells and in vivo phenotypic manifestations of OPA-deletion on the spinal column, and knee joint in *Opa1*cKO mice.

Furthermore, H&E staining showed synovial hyperplasia/ossification in the lateral and medial knee compartments of 20-month-old *Opa1cKO* mice (Fig. 10A) however, there were no morphological changes between WT and *Opa1cKO* mice at 12 months (Fig. S6. F, G). Additionally, we conducted histomorphometric analysis on both lateral and medial knee joint compartments of 12 and 20-month-old mice. In the lateral compartment of 20-month *Opa1*cKO mice there was a significant decrease in the area and thickness of articular cartilage and calcified cartilage (Fig. 10B, B’, C, C’, D, D’). SCBP area and thickness were significantly increased in *Opa1cKO* mice when compared to controls, indicating enhanced subchondral bone sclerosis in OPA1 loss mice, a finding that aligns with our μCT data (Fig. 10D, D’). We also noted a considerable increase in the synovial hyperplasia/ossification score in the lateral joint compartment of 20-month-old *Opa1cKO* mice (Fig. 10E). On the other hand, 12-month-old mice showed no significant morphological and histomorphometric changes or evidence of an OA phenotype (Fig. S6F-M’). It is interesting to note that the profound OA phenotype in *Opa1cKO* mice was primarily observed in the lateral compartment as opposed to the medial compartment which is typically more affected. These findings demonstrate that cartilage-specific loss of OPA1 enhances the severity of age-associated OA in mice.

## DISCUSSION

This study for the first time establishes a causal relationship between sustaining mitochondrial dynamics through OPA1 and maintenance of the spine and knee joint health during aging in mice. The reliance on mitochondrial function was notable since hypoxic chondrocytes and in particular NP cells of IVD primarily rely on glycolysis for energy production^5,6^. Relevant to this finding, we have previously shown the existence of mitochondrial networks in NP cells (7) and that mitochondrial activity is governed by the HIF-1α-BNIP3 axis controlling mitophagic flux^6^. We also demonstrated that deletion of mitophagy receptor BNIP3 in NP cells caused mitochondrial dysfunction affecting bioenergetics, triggering meta-inflammation, and resulting in early disc degeneration in mice^5^. Moreover, our transcriptomic analyses of human NP tissues showed an association of biological themes related to mitochondrial functions with disc degeneration^11^. Herein, we show that, OPA1 controls the morphology of mitochondria and cristae as well as multiple organelles including peroxisomes, early endosomes, and cis-Golgi, and that OPA1-loss results in dysregulated autophagy in NP cells. Further, we demonstrate that *Opa1*^*AcanCreERT2*^ mice evidence accelerated age-dependent IVD degeneration, vertebral osteopenia, and severe OA of knee joints. Our findings highlight the fact that dysregulation of mitochondrial dynamics affects the metabolism, organelle integrity, and the autophagic/mitophagic pathway causing disc and cartilage degeneration in mice.

OPA1 is required for IMM fusion as well as the maintenance of cristae shape and its mutations are linked to multiple pathologies including vision impairment^28^, developmental delay, muscle-related disorders, peripheral neuropathy, and cardiomyopathy^29^. Concerning axial skeleton, we have previously showed that changes in mitochondrial morphology impact NP cell metabolism and mitophagy and pathways related to mitochondrial dysfunction are enriched in transcriptomes of degenerated human NP tissues^5,6^. Herein we observed that deletion of OPA1 resulted in a reduction in mitochondria size but an increase in numbers. This change was unlikely due to mitochondrial biogenesis since there was no difference in the mtDNA content of the mutated cells.^30,31^. More than likely, as the OPA1-deficient NP cells lacked cristae or exhibited detached cristae it indicated that OPA1 was required for IMM invagination and cristae development. Moreover, it is known that mitochondria interact with other organelles and maintain cellular homeostasis while maintaining mitochondrial function^32^. From this perspective, impact on peroxisomes, endosomes, and cis-Golgi morphology suggested that OPA1 is critical for preserving multi-organelle morphology likely through governing their interactions with mitochondria in NP cells. To the best of our knowledge, this is the first report on the involvement of OPA1 in sustaining the morphology of these organelles.

When mitochondrial quality control was assessed, a significant decrease in key autophagy-related proteins p62 and LC3-II in OPA1-knockdown cells was noted. Moreover, Bafilomycin A_1_ treatment showed a lack of LC3-II accumulation suggesting impaired autophagosome formation. LC3-II targets ubiquitin-positive cargo to sequester them into developing autophagosomes^33^. Notably, these autophagy proteins are subjected to regulatory post-translational phosphorylation by mTORC1 (negative) and ULK1 and AMPK (positive) whereby AMPK promotes autophagy by phosphorylating ULK1 when the ADP/ATP or AMP/ATP ratio is elevated. Indeed, our metabolic profiling indicated that decreased AMP/ATP and ADP/ATP ratios would be associated with decreased phosphorylation, and, as a consequence, reduced autophagy. In contrast to previous studies demonstrating enhanced mitophagy in OPA1 defective cells^31,34,35^, we noted that OPA1-knockdown not only inhibited selective autophagy (mitophagy) but also macroautophagy, an event that influenced the morphology of multiple organelles, including the ER, endosomes, and Golgi^36–38^. For example, enlarged early endosomes and fragmented Golgi, together with autophagosome accumulation is implicated in neurodegenerative diseases such as Down syndrome and Parkinson’s,^39,40^. Importantly, changes in these organelles underscore the defects in autophagy and disruptions in endocytic and secretory pathways in OPA1-deficient NP cells. Together, our data supports the hypothesis that OPA1 regulates organelle morphology and autophagic/endocytic/secretory pathways in NP cells. One important outcome of these findings is that autophagy and mitochondrial dysfunction are associated with aging, disc degeneration, and the pathogenesis of OA^2,41,42^.

Considering the relationship between OPA1 and mitochondrial function, it is known that in many cell types, which primarily rely on oxidative ATP generation, structural changes in mitochondria profoundly alter energy metabolism. However, since NP cells are predominantly glycolytic, it was important to determine whether alterations in mitochondrial shape and cristae morphology influenced NP cell bioenergetics. We found that while fragmented mitochondria with aberrant cristae morphology consumed more oxygen, it did not manifest in increased oxidative ATP production rates. The number of ATP molecules generated for each dioxygen molecule consumed might vary depending on the mitochondrial efficacy^43,44^, suggesting mitochondrial dysfunction in the Sh*Opa1* transduced NP cells. Our findings also differed from prior work showing lower OCR and oxidative ATP generation in OPA1-deficient cells^45,46^ but were in agreement with the increase in glycolytic ATP production rate noted in OPA1-deficient neutrophils^47^.Overall, the results of the current study underscored the observation that OPA1 deficiency influences the energy metabolism of NP cells.

In addition to bioenergetics, the mitochondrion is also the primary site for biosynthetic pathways. Metabolic systems in OPA1-deficient NP cells that were most negatively impacted involved spermidine and spermine biosynthesis and betaine metabolism. Likewise, there was a decrease in amino acid metabolism, taurine and hypotaurine synthesis, pyrimidine, pantothenate and CoA biosynthesis, and urea cycle metabolite. In agreement with these findings, OPA1 deletion in MEF cells resulted in decreased levels of metabolites associated with spermidine and spermine and taurine and hypotaurine pathways^48^. Notably, spermidine and taurine levels are shown to decline with age, and mitochondrial dysfunction is one of the primary contributors to their deficiency^49,50^. Supplementation of spermidine and taurine has been proven to prolong longevity in mice via boosting autophagy/mitophagy, mitochondrial biogenesis, and mitochondrial respiration^49,50^. Interestingly, the increased levels of glucose-6-phosphate was, not attributable to enhanced glycolysis associated with the lack of corresponding increase in intracellular and extracellular lactate levels. Depending on cellular metabolism, glucose-6-phosphate shuttles between glycolysis, pentose phosphate shunt, hexosamine biosynthetic pathways, gluconeogenesis, and de-novo lipid synthesis^51^. Accordingly, in Sh*Opa1* cells, glucose-6-phosphate was utilized for the synthesis of NADP, nucleotide sugar metabolism, and gluconeogenesis pathway activity. Furthermore, elevated malate and the increased malate-aspartate shuttle activity are linked to gluconeogenesis, while increased serine levels in Sh*Opa1* cells indicated de novo serine synthesis, implying that malate was exported to the cytosol^52,53^. Interestingly, acetylcarnitine level was decreased in OPA1-deficient NP cells, which links mitochondrial metabolism to histone acetylation and lipogenesis^54^. In agreement with a previous report showing OPA1 regulation of lipid metabolism, we also observed reduced fatty acid metabolism^55^. OPA1 deficiency also affected one-carbon metabolism with increased serine biosynthesis but decreased utilization in ShOpa1 cells. This reduction was likely due to a decrease of s-adenosyl homocysteine and s-adenosyl methionine co-substrates, which are involved in transferring methyl groups for the synthesis of DNA, amino acids, and polyamines. This was further supported by reduced pyrimidine metabolism, polyamines, and amino acids metabolism. Notably, serine biosynthesis and one-carbon metabolism are linked to a variety of mitochondrial disorders^56^. In summary, the metabolite profiling studies revealed broad metabolic dysregulation in OPA1-deficient NP cells.

^13^C isotope labeling experiments utilizing two stable isotope tracers [1,2]-^13^C-glucose and U^13^C-glutamine shed further insights into metabolic dysregulation of OPA1-deficient NP cells. The use of [1,2]-^13^C-glucose MFA revealed a reduction in PDH flux despite an increase in alanine ∑ mn indicating an elevated pyruvate pool. We also noted a decrease in intracellular M + 1 glutamate enrichment, an indirect measurement for alpha-ketoglutarate, suggesting pyruvate is prevented from entering the TCA cycle via the conventional pathway. Furthermore, since there was no change in PC flux, this supports the anaplerosis i.e. pyruvate carboxylation replenishes TCA intermediates and allows the Krebs cycle to continue since the intermediates are not only important for macromolecule synthesis but also for protein post-translational modifications, chromatin modification, and DNA methylation^57^. Interestingly, in solid hypoxic tumors, increased anaplerosis through PC is required for extracellular collagen production and aberrant fibrosis by tumor-associated fibroblasts, a phenotype we have noted in the discs of *Opa1*cKO mice^58^. Regarding glutamine utilization, the labeled glutamine entered the TCA via an anaplerotic reaction to alpha-ketoglutarate with M + 4 labeling of the forward TCA cycle intermediates succinate, fumarate, malate, and citrate. In contrast to glucose, glutamine labeling showed decreased TCA cycle intermediates such as citrate, succinate, fumarate, malate, and oxaloacetate (aspartate) enrichment. The Citrate M + 4 label signifies the oxidative (forward) TCA cycle while the M + 5 label is for the reductive (reverse) TCA cycle. We noted a substantial reduction in citrate M + 5 label (~ 2.5:1 M + 4:M + 5 citrate), indicating a modest reversal in TCA flux. Of interest, under normal physiological conditions, the majority of succinate is generated by oxidative or forward TCA cycle. Recent studies found that under hypoxic conditions a part of succinate was generated from fumarate or when ETC was inhibited, implying that fumarate reduction to succinate may act as a valve for surplus electrons from the ETC. Further, it was reported that fumarate was catalyzed explicitly by complex II, rather than passively collecting leaky electrons from the ETC^23^. Interestingly, our ^13^C MFA data showed increased succinate ∑ mn compared to fumarate in both ShCtrl and Sh*Opa1* cells, suggesting an oxidative TCA cycle. Furthermore, when we assessed succinate oxidation (fumarate M + 4/succinate M + 4) and fumarate reduction (succinate M + 3/fumarate M + 3), we observed no changes in the oxidative process. Overall, these findings suggested that NP cells do not experience metabolic stress by their physiologically hypoxic niche. These studies suggested that mitochondrial morphology and cristae architecture are not only central to energy metabolism, but the integrity of these structures is critical for the proper functioning of mitochondria in NP cells.

Finally, we investigated the *in vivo* function of OPA1 in the IVD and knee cartilage. We found that *Opa1cKO* mice evidenced enhanced IVD degeneration with aging. Interestingly, the caudal discs showed a more pronounced phenotype than the lumbar discs. Previous studies have shown that caudal discs of mice are more prone to metabolic dysregulation and subsequent degeneration with aging^59^. Moreover, the caudal spine experiences relatively lower axial loading and different motions than the lumbar spine, it is therefore not unreasonable to hypothesize that the unique interactions between genetics and environmental factors produce varying phenotypic outcomes across different spine regions^60^. Similar to IVD degeneration, aged *Opa1*cKO displayed enhanced age-associated OA severity; the disease state was characterized by enhanced cartilage damage, osteophyte formation, subchondral bone sclerosis and synovial hyperplasia and/or ossification. While the medial compartment of the knee is usually prone to cartilage degeneration^61^, interestingly, the lateral knee compartment in *Opa1cKO* mice exhibited significantly more pronounced OA. Similar to caudal discs, the lateral knee compartment experiences lower compressive loads^62^. Moreover, consistent with previous studies that demonstrate exercise and/or loading in human and animal models can partially restore the functionality of faulty mitochondria^63,64^, lack of substantial degeneration in lumbar discs and the medial compartment of the knee suggests a protective effect of loading on these joint tissues^60,65^. Regarding the age-dependency of the phenotypes, of course, in hypoxic skeletal tissues, chronic metabolic stress arising from abnormalities in mitochondrial function would be expected to influence tissue function, especially in aging individuals.

It is important to note that similar to the NP and knee cartilage, significant degenerative changes affect the AF in *Opa1*cKO mice, underscoring the important contribution of mitochondrial activity to annulus tissue health. This observation is supported by a previously reported microarray analysis of human AF tissues that showed altered genes related to mitochondrial function during degeneration^66^. Moreover, similar to OA development, in degenerated AF tissues, ROS-related gene expression is dramatically altered, suggesting that mitochondrial dysfunction and ROS production promote AF degeneration^66^. These *in vivo* findings coupled with our mechanistic studies suggest that the mouse IVD, spinal column, and joint cartilage phenotypes is the outcome of metabolic dysregulation and cumulative degenerative processes driven by OPA1 deletion. Moreover, in many soft tissues, age-related pathologies, and metabolic disorders due to the accumulation of faulty mitochondria has been exhaustively demonstrated^67,68^. From this perspective, targeting and modifying the autophagic pathway and preserving mitochondrial function should be of major concern when designing therapeutics to treat diseases linked to degenerative musculoskeletal conditions.

## MATERIALS AND METHODS

### Cell isolation and treatments

Primary NP cells were isolated from adult Sprague Dawley rats (Charles River) and cultured in antibiotic supplemented low glucose (1g/L) containing DMEM and 10% FBS^69^. For lentiviral particle production and viral transduction LV-Sh*Opa1* clone #1 (TRCN0000091111) and LV-SH*Opa1* clone #2 (TRCN0000348537), pLKO.1 ShCtrl (Sigma, St. Louis, MO, USA) and packaging vectors psPAX2 (#12260) and pMD2G (#12259) (Addgene, Cambridge, MA, USA) were used. 1 day before transfection, HEK 293T cells were plated in 10-cm plates (5×10^6^ cells/plate) in DMEM with 10% heat-inactivated FBS. ShCtrl or Sh*Opa1* plasmids, as well as psPAX2 and pMD2.G, were transfected into cells and lentiviral particles were collected 48 to 60 hours after transfection. Passage 2 NP cells were transduced with viral particles using 8 mg/ mL polybrene and after 3 days of transduction cell were cultured hypoxia workstation (Invivo2 400; Baker Ruskinn, Bridgend, UK) with a mixture of 1% O_2_, 5% CO_2_, and 94% N2 for 24 h and on day 5 cells were harvested for protein extraction. Since both OPA1 shRNA clones showed similar effects on mitochondrial morphology and mitophagy, cells transduced with LV-Sh*Opa1* #1 (TRCN0000091111) were utilized for all metabolic investigations.

### Immunocytochemistry

ShCtrl, Sh*Opa1* or DFP treated NP cells were plated on glass coverslips and treated with 100 nM MitoTracker Red CMXRos (Thermo Fisher Scientific, Waltham, MA, USA; M7512) for 30 minutes after completion of the experimental treatments. Cells were then fixed with 4% PFA or ice-cold methanol for 15 minutes and permeabilized with 0.1% Triton X-100 for 10 minutes and blocked with 1% BSA for 1 h. Cells were incubated with anti-OPA1 (612607) (BD Bioscience), anti-PMP70 (PA1–650), anti-GM130 (MA5–35107) (Fisher Scientific, Pittsburgh, PA, USA), anti-EEA1 (24115), anti-BNIP3 (3769S), anti-LC3B (12741) (Cell signaling, Denver, MA, USA), anti-TNG46 (ab16059), anti-LAMP1 (ab24170), and anti-BNIP3L (ab109414), (Abcam, Cambridge, MA, USA), and anti-RAB7 (R8779) (EMD Millipore, Burlington, MA, USA), in blocking buffer at 1:100 to 200 at 4 °C overnight. After washing, cells were incubated with Alexa Fluor 488 and mounted with ProLong Gold Antifade Mountant with DAPI. The specificity of staining cells was confirmed using isotype mouse (7076P2) or rabbit (7074P2) IgG antibodies (Cell Signaling, Danvers, MA, USA). Cells were visualized using a Zeiss LSM 800 Axio Inverted confocal microscope (Plan-Apochromat 40x/1.3 oil or 63x/1.40 oil) for positive staining for markers OPA1, LC3, PMP70, EEA1, GM130, TGN46, RAB7, LAMP1, BNIP3 and BNIP3L.

### Organelles morphology analysis

Mitochondria, peroxisomes, endosomes and Golgi number, branching and morphology were quantified in ImageJ using methods reported earlier^5,6^. Briefly, the confocal images were converted to binary by threshold and then converted to a skeleton that represented the features in the original image using a wireframe of lines one pixel wide. All pixels within a skeleton were then measured using analyze skeleton. The output will give the number of particles which denotes the total number of mitochondria, the aspect ratio (AR) represents the “length to width ratio” and the form factor (FF), the complexity and branching aspect of mitochondria were calculated from circularity. For the endosome, Feret diameter was used to plot the graph.

### Western Blotting

ShCtrl, ShOpa1 transduced cells were lysed and 35 μg of protein was electroblotted to PVDF membranes (EMD Millipore, Burlington, MA, USA). The membranes were blocked and incubated overnight at 4°C with antibodies against anti-BNIP3 (3769), anti-BNIP3L (12396), anti-LC3B (2775), anti-LAMP1 (9091), anti-p62 (5114), anti-CHOP (78063), anit-Beclin1 (3738), anti-MFF (84580), anti-MFN2 (9482), anti-FIS1 (32525), anti-Ub (#), pUb (62802), anti-GAPDH (5174) (Cell Signaling), anti-DRP1 (611113), anti-OPA1 (612607) (BD Biosciences, San Jose, CA, USA), anti-PARKIN (sc-32282), anti-MFN1 (ab126575) (Abcam, Cambridge, MA, USA), Immunolabeling was detected using an ECL reagent (Azure biosystems 300, Dublin, CA, USA). The membranes were detected using ECL reagent (Azure biosystems, Dublin, CA, USA). Densitometric analysis was performed using ImageJ software.

### Seahorse XF analysis

Maximum glycolytic capacity and ATP production rate using methods reported by Mookerjee and colleagues^5,21,22^. In brief, ShCtrl, Sh*Opa1* cells were plated in a 24-well Seahorse V7-PS test plate under hypoxia 24 hours before the experiment. Cells were washed three times with 500 μl of KRPH (Krebs Ringer Phosphate HEPES) before being cultured for one hour at 37 °C in 100% air. For glycolytic capacity calculation, oxygen consumption rate (OCR) and related extracellular acidification rate (ECAR) were determined in a Seahorse XFe24 analyzer (Agilent Techonoligies) by adding 10 mM glucose, 1 μM rotenone plus 1 μM myxothiazol, and 200 μm monensin plus 1 μM FCCP via ports A-C. OCR and ECAR were assessed by adding 10 mM glucose, 2 μg oligomycin, 1 μM rotenone plus 1 μM myxothiazol to determine ATP generation rate from oxidative and glycolytic pathways.

### Widely targeted small metabolite measurements

Cells were transduced with ShCtrl and ShOpa1 viral particles as described above. On the third day, the medium was changed to DMEM without pyruvate, 10% dialyzed FBS (Sigma F0392), and the cells were grown under hypoxia for 24 hours. Cells were washed and collected in ice-cold 80% methanol before being snap-frozen in liquid nitrogen and kept at −80 degrees Celsius until use. Prior to analyzing the metabolites, cell pellet samples were centrifuged and pipetted into a LC sampling vial. Each sample had internal standards. After drying under mild nitrogen flow, the samples were reconstituted in 150 μl of 80% methanol for injection. The samples were analyzed on an ABsciex 6500 + coupled with a Waters UPLC. Small metabolites were separated using the Ace PFP column and the iHILIC-p column (HILICON) and a pooled quality control (QC) sample was added to the sample list. The QCs sample was injected six times to calculate the coefficient of variation (CV) for data quality control. Metabolites with CVs lower than 30% used for the quantification. MetaboAnalyst 5.0 web server was used to analyze the data, and acceptable metabolites were manually input using the HMDB number. The small metabolites pathway data bank (SMPDB), which contains 99 compounds based on normal human metabolic pathways, was used for enrichment and pathway analysis. MetaboAnalyst provides the list of pathways in which these metabolites are found.

#### ^13^ C-Metabolic flux analysis

For [1,2]-^13^C-glucose flux analysis, 50% of DMEM media contained ^13^C-labeled glucose. A volume of 100 μl of cell culture medium from 1,2-^13^C glucose experiment was treated with 400 μl of methanol. After centrifugation, the supernatant was transferred to a LC-MS sampling vial and dried under gentle nitrogen flow. The sample was reconstituted into 100 ml of 80% methanol for LC-MS injection. Metabolite separation was performed on an ACE PFP-C18 column (1.7 μM × 1 mm × 100 mm) and analyzed on a ABSciex 6500 + with a Multiple Reaction Monitoring (MRM) mode. The glycolysis, pentose cycle, PDH, PC, PDH/PC, and PDH + PC fluxes were calculated using methods reported by Madhu et al.,^5^.

When flux was assessed in the U^13^C-glutamine labeling experiment, labeled glutamine was added to be 50% of the total DMEM glutamine concentration. Methanol extraction from [1,2]-^13^C-glucose and U^13^C glutamine labeled cell pellets were dried under gentle nitrogen flow. The dried samples were derivatized with a methyl-moximation (with 15 mg/ml methoxy amine in pyridine, 30 °C for 90 minutes) and MTBSTFA (at 70°C for 60 minutes). The samples were then analyzed with an Agilent GC-MS, with an electron impact mode and a DB-5MS column (Agilent) following our protocol^5^. The data were analyzed with Mass Hunter Quantitative Analysis software (Agilent). The enrichment was calculated after subtraction from the background of the non-labeled treatment samples. Fractional enrichments from G to R is sigma (stands for sigma mean), and it is equal to the weighted mean average of the metabolite’s enrichment (sigma mn = 1 xm1 + 2xm2 + 3xm3, etc).

### Mitochondrial DNA quantification

DNA was isolated from ShCtrl and Sh*Opa1* cells using DNA extraction lysis buffer (Qiagen). The mitochondrial DNA (mtDNA) content was determined using qPCR (Applied Biosystems A25742 PowerUP SYBR green master mix). For mitochondrial DNA (mtDNA; Nd1) 5’-GGC TCC TTC TCC CTA CAA ATA C-3’ and 5’-TGT TTC TGC AAG GGT TGA AAT G-3’. For nuclear DNA (nDNA; Cox4) 5’-ATG TTG ATC GGC GTG ACT AC-3’ and 5’-AGT GGG CCT TCT CCT TCT-3’ were used. The ratio of mtDNA to nDNA (mtDNA/nDNA) reflects the relative mtDNA content.

### Generation of conditional knockout mice

All mouse studies were carried out in conformity with the Institutional Animal Care and Use Committee (IACUC) of Thomas Jefferson University’s applicable rules and regulations. *Opa1*^*fl/fl*^ on mixed C57BL/6–129/SvEv background were described previously^46^. Aggrecan-CreERT2 mice were from Jackson Laboratories (stock #019148). OPA1 conditional knock-out (*Opa1cKO:Acan*^*CreERT2*^*Opa1*^*fl/fl*^) and control (*Opa1*CTR: *Opa1*^*fl/fl*^) mice were generated and analyzed after 3 (7-month-old), 9 (12-month-old), and 17 (20-month-old) months after tamoxifen injections to determine degree of degeneration. For all experiments, skeletally mature 3-month-old female and male mice of all genotypes received an intraperitoneal injection of 100 mg/kg tamoxifen (Sigma-Aldrich, St. Louis, MO, USA) dissolved in palm oil (Sigma-Aldrich) for 3 consecutive days to activate Cre recombinase.

### Histological analysis of intervertebral disc

Spines were dissected and immediately fixed in freshly made 4% paraformaldehyde (PFA) for 48 hours, followed by decalcification in 20% EDTA at 4°C prior to embedding in paraffin. Lumbar and caudal motion segments from 7-month-old (6–7 mice/genotype, 25–30 lumbar and 12–14 caudal discs/genotype), 12-month-old (8–9 mice/genotype, 32–36 lumbar and 18–19 caudal discs/genotype) 20-month-old (9–11 animals/genotype, 36–44 lumbar and 27–33 caudal discs/genotype) WT and *Opa1cKO* mice were processed. 7 μm coronal sections were stained with 1% SafraninO, 0.05% Fast Green, and 1% Hematoxylin and imaged on an Axio Imager A2 microscope using 5x/0.15 N-Achroplan or 20x/0.5 EC Plan-Neofluar (Carl Zeiss) objectives, Axiocam 105 color camera, and Zen2^™^ software (Carl Zeiss AG, Germany). 4 blinded observers used a Modified Thompson grading scale for the NP and AF compartments to score histological sections^11,41,59^. Picrosirius red staining (Polysciences, 24901) was used to measure collagen fibril thickness, and pictures were captured using a polarizing light microscope (Eclipse LV100 POL; Nikon) and a 4x /0.25 Pol /WD 7.0 (Nikon) objective. The color thresholds for green (thin), yellow (middle), and red (thick) fibers were established using the Nikon NIS Elements Viewer program^26,70^. For all samples, the color threshold values remained constant. Since phenotypic outcome at each of the spinal level is governed by the unique interactions between the environmental (mechanical), biological and genetic factors, each disc was considered as a unique sample^11,41,59^.

### Immunohistochemistry and confocal analysis

Mid-coronal 7 μm thick disc tissue sections were de-paraffinized and incubated in microwaved citrate buffer for 20 min, proteinase K for 10 min at room temperature, or Chondroitinase ABC for 30 min at 37°C for antigen retrieval. Sections were blocked in 5% normal serum (Thermo Fisher Scientific, 10000 C) in PBS-T (0.4% Triton X-100 in PBS) and incubated with antibody against GLUT-1 (1:200, Abcam, ab40084), CA3 (1:150; Santa Cruz Biotechnology, sc-50715) Collagen X (1:500, Abcam, ab58632) Collagen I (1:100; Abcam, ab34710), COMP (1:200, Abcam, ab231977), ACAN (1:50; Millipore Sigma, AB1031), ARGXX. (1:200; Abcam, ab3773). When using mouse primary antibodies, Mouse on Mouse Kit (Vector laboratories, BMK2202) was used for blocking during primary antibody incubation. Tissue sections were thoroughly washed and incubated with Alexa Fluor - 488 or 594 or 647 conjugated secondary antibodies (Jackson ImmunoResearch Lab, Inc.), at a dilution of 1:700 for 1 h at room temperature in dark. The sections were washed with PBS-T (0.4% Triton X-100 in PBS) and mounted with ProLong^®^ Gold Antifade Mountant with DAPI (Thermo Fisher Scientific, P36934). All mounted slides were visualized using a Zeiss LSM 800 Axio Inverted confocal microscope (Plan-Apochromat 5x/0.16 or 10x/0.45). ImageJ 1.52i (NIH) was used for all quantifications. Images were threshold to generate binary images, then NP and AF compartments were contoured manually using the Freehand Tool. The ROI were examined using the Area Fraction measurement.

### Micro-computed tomography (μCT) analyses

μCT imaging was performed on the lumbar spine of 7, 12, and 20-month WT and Opa1cKO mice using the high-resolution μCT scanner (Skyscan 1272, Bruker, Belgium). Lumber segments L2–6 were placed in PBS and scanned with an energy of 50 kVp and current of 200 mA resulting in 15 mm^3^ voxel size resolution. Trabecular parameters were assessed in the 3D reconstructed trabecular tissue using Skyscan CT analysis (CTAn) software by contouring the region of interest (ROI). The bone volume percentage (BV/TV), trabecular number (Tb. N.), trabecular thickness (Tb. Th.), and trabecular separation (Tb. Sp.) of the resulting datasets were all evaluated. Cortical bone volume (BV), cross-sectional thickness (Cs. Th.), mean cross-sectional bone area (B. Ar), and mean cross-sectional tissue area (T. Ar) were all measured in two dimensions. A standard curve was established with a mineral density calibration phantom pair (0.25 g/cm3 CaHA and 0.75 g/cm3 CaHA) to determine mineral density. Intervertebral disc height and the length of the vertebral bones were measured and averaged along the dorsal, midline, and ventral regions in the sagittal plane. Disc height index (DHI) was calculated as previously described^26,70^.

Mouse hindlimbs (right limb) were harvested, surrounding muscles were removed, and limbs were fixed in formalin (10%, 48 hours). MicroCT analysis of the femur, tibia, and knee joint were performed on a Bruker SkyScan 1275 scanner as we have previously described^27^. MicroCT reconstructions and quantitative analysis of tibial subchondral bone volume fraction (BV/TV), trabecular thickness (Tb.Th), trabecular separation (Tb.Sp), and subchondral bone plate thickness (SCBP) were performed using the SkyScan CT Analyzer (CTan) and CT Vox software on coronal slices that spanned the medial and lateral tibial plateaus.

### Histological and histomorphometry analysis of OA

Hindlimbs were decalcified (EDTA, 19%, 21 days), processed, paraffin embedded, and sectioned along the coronal plane, as we have described^27^. Mid-coronal sections were stained with hematoxylin and eosin (H&E), or toluidine blue and OA severity was analyzed by Articular Cartilage Structure (ACS), toluidine blue, and osteophyte scoring on the medial and lateral tibial plateaus (MTP, LTP) and femoral condyles (MFC, LFC). Synovial hyperplasia was assessed using a 0–3 scale as described earlier^71^ In joints that presented with synovial ossification, a maximal synovial hyperplasia score was assigned. Analysis was performed by a blinded scorer with experience of the OA scoring techniques. Detailed histomorphometric analysis of articular cartilage thickness and area, calcified cartilage thickness and area, and subchondral bone thickness and area were analyzed on the MTP and LTP using ImageJ software as we have previously described^27^.

### Statistical analysis

Statistical analysis was performed using Prism9 (GraphPad, La Jolla, CA, USA). The quantitative data are represented as mean ± SEM or Box and whisker plots showing all data points with median and interquartile range and maximum and minimum values. Data distribution was checked with Shapiro-Wilk normality test, and differences between two groups were assessed by t-test or Mann-Whitney test as appropriate. One-way ANOVA, whereas non-normally distributed data were analyzed using or Kruskal Wallis test with appropriate post-hoc test (Sidak’s multiple comparisons test) was used for comparisons between more than two groups. Analysis of Modified Thompson Grading data distribution and fiber and fiber thickness distribution were performed using chi-square test; p < 0.05.

## Data Availability

All data generated or analyzed during this study are included in this article. Additional raw data files can be obtained from the corresponding author upon request.
